# Sense and antisense *OsDof12 *transcripts in rice

**DOI:** 10.1186/1471-2199-9-80

**Published:** 2008-09-17

**Authors:** Dejun Li, Chunhua Yang, Xiaobing Li, Guobiao Ji, Lihuang Zhu

**Affiliations:** 1 State Key Laboratory of Plant Genomics & National Plant Gene Research Center (Beijing), Institute of Genetics and Developmental Biology, Chinese Academy of Sciences, 5 Datun Road, Chaoyang District, Beijing 100101, PR China; 2 Graduate School of Chinese Academy of Sciences, Beijing 100049, PR China

## Abstract

**Background:**

Antisense transcription is a widespread phenomenon in plants and mammals. Our previous data on rice gene expression analysis by microarray indicated that the sense and antisense transcripts at the *OsDof12 *locus were co-expressed in leaves. In current study, we analyzed the expression patterns in detail and looked for the possible mechanism related to their expression patterns.

**Results:**

*OsDof12*, being a single copy gene located on rice chromosome 3, encodes a predicted Dof protein of 440 amino acids with one intron of 945 bp. The antisense transcript, *OsDofl2os*, overlaps with both the exonic and intronic regions of *OsDof12 *and encodes a functionally unknown protein of 104 amino acids with no intron. The sense-antisense *OsDof12 *transcripts were co-expressed within the same tissues, and their expressions were not tissue-specific in general. At different developmental stages in rice, the *OsDof12 *and *OsDof12os *transcripts exhibited reciprocal expression patterns. Interestingly, the expression of both genes was significantly induced under drought treatment, and inhibited by dark treatment. In the *Pro*_*OsDof*12_*-GUS *and *Pro*_*OsDof*12*os*_-*GUS *transgenic rice plants, the expression profiles of GUS were consistent with those of the *OsDof12 *and *OsDof12os *transcripts, respectively. In addition, the analysis of cis-regulatory elements indicated that either of the two promoters contained 74 classes of cis-regulatory elements predicted, of which the two promoter regions shared 53 classes.

**Conclusion:**

Based on the expression profiles of *OsDof12 *and *OsDof12os*, the expression patterns of GUS in the *Pro*_*OsDof*12_*-GUS *and *Pro*_*OsDof*12*os*_-*GUS *transgenic rice plants and the predicted common cis-regulatory elements shared by the two promoters, we suggest that the co-expression patterns of *OsDof12 *and *OsDof12os *might be attributed to the basically common nature of the two promoters.

## Background

The gene regulation by natural antisense RNA in prokaryotes has been known for many years [1,2]. The first example was found in the plasmid ColE1, in which DNA replication was regulated by an antisense RNA [3,4]. Later, the natural antisense RNAs involved in the regulation of gene expression were also identified in a number of eukaryotes [5,6] including plants [7] and animals [8]. In the past few years, many regulatory RNA molecules have been characterized in eukaryotes [9,10]; one class of such regulatory RNA is the natural antisense transcripts (NATs). In general, the sense strand of a genomic locus acts as a template for production of mRNA, but the mRNA may have its endogenous antisense RNA transcribed from the opposite strand. NATs are a class of endogenous coding or non-coding RNAs that have sequence complementarity to other RNAs in the cell. According to the genomic location of the two DNA strands that generate sense and antisense transcripts, respectively, NATs can be divided into cis-NATs, which are transcribed from opposing DNA strands at the same genomic locus, and *trans*-NATs, which are transcribed from separate loci. cis-NAT pairs display perfect sequence complementarity (as expected from their genomic overlap), whereas *trans*-NAT pairs display imperfect complementarity. Due to the genomic location with sense transcripts, most natural antisense transcripts reported so far are cis-NATs [5,6].

Genome-wide identification of antisense transcripts in several model organisms, including human, mouse, Drosophila, Arabidopsis and rice, has revealed the widespread existence of NATs [11-20]. In Arabidopsis, Yamada et al. [17] reported that about 30% of all annotated genes showed significant antisense RNA expression; later Wang et al. [18] predicted 1,340 potential NAT pairs with a new computational method. In rice, the RIKEN group [19] uncovered 687 bi-directional transcript pairs from 32,127 full-length cDNA sequences and mRNA sequences; In addition, 23.8% of rice transcripts were identified that showed antisense RNA expression by high-density oligonucleotide tilling microarray analysis [20].

Although a large amount of sense-antisense transcript pairs have been predicted or identified in plants, only three of them have been systematically analyzed in their expression modes or regulatory mechanisms in detail [21-25]. In *Petunia hybrida*, the 3' region of the *Sho *gene contains a promoter in the opposite orientation that produces a partially overlapping antisense transcript. The antisense transcription can be activated in a tissue-specific manner to adjust local cytokinin synthesis via degradation of *Sho *dsRNA [21]. In *Arabidopsis*, salt tolerance is regulated by two small interfering RNAs (siRNAs) produced from a pair of tail-to-tail overlapping protein-encoding genes, *P5CDH *(a stress-related gene) and *SRO5 *that is induced by the salt treatment. When the two genes are transcribed, a RNA duplex is formed and siRNAs are produced, which can ultimately cleave the *P5CDH *transcripts [22]. In the third case reported by Katiyar-Agarwal et al., a type of endogenous siRNA, nat-siRNAATGB2, can be specifically induced by the bacterial pathogen, *Pseudomonas syringae*, carrying effector *avrRpt2*. This siRNA contributes to *RPS2*-mediated race-specific disease resistance by repressing PPRL, a proposed negative regulator in the *RPS2 *resistance pathway [23,24]. Analyzing the expressions of sense and antisense partners and identifying the siRNAs that match these cis-NATs, Jin et al. suggest that siRNA regulation of cis-NATs via the RNAi pathway is an important gene regulatory mechanism for at least a subgroup of cis-NATs in Arabidopsis [25].

Analyzing rice gene expression using single-strand oligo microarray, we detected the expression of an antisense transcript at a Dof gene locus (data not shown), which was also reported as antisense transcript in a global survey of the rice antisense transcripts by Osato et al. [19]. The Dof gene we studied had been named as *OsDof12 *[26]. The sense transcript of the sense-antisense transcript pair, *OsDof12*, encodes a Dof transcriptional factor while the antisense transcript, *OsDof12os*, represents a function unknown gene. In current study, we characterized the structures and expression patterns of sense-antisense *OsDof12 *transcripts in detail. In addition, possible mechanisms related to their expression patterns and antisense regulation were discussed.

## Results

### *OsDof12 *is a single copy gene located on rice chromosome 3

**Figure 1:**
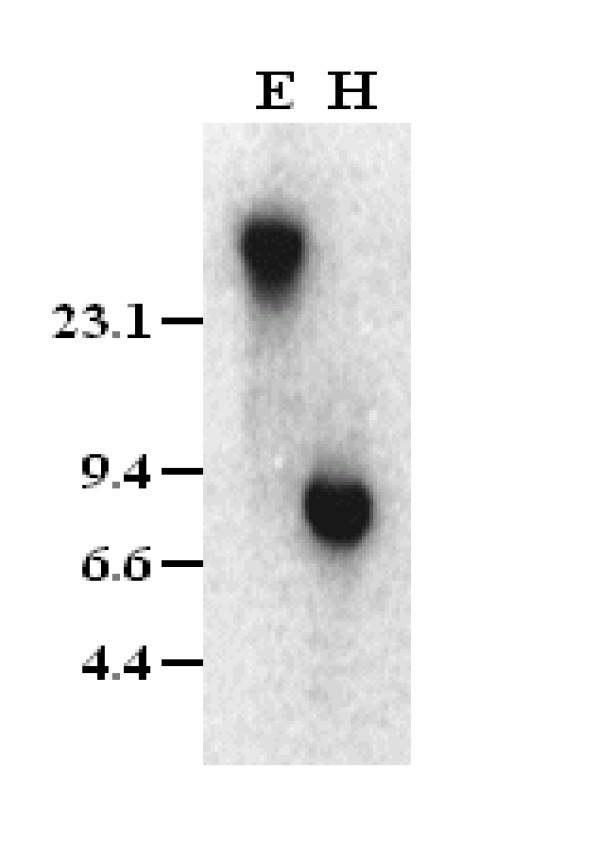
**Southern blotting analysis of *OsDof12***. Single band was detected in genomic DNA digested with HindIII(H) or EcoRI(E) using probe C. A total of 10 μg Lambda DNA (Promega) was digested with HindIII and served as the molecular length marker.

BLAST results show that the *OsDof12 *gene is a single copy gene located on rice chromosome 3 [27]. To further confirm this, total DNA from rice leaves was extracted and digested with HindIII and EcoRI, respectively, and analyzed by Southern blotting under stringent conditions. To prevent cross hybridization, we used the *OsDof12 *specific probe (probe C) that spans the segment from 670 bp to 1378 bp in the *OsDof12 *full-length cDNA. As shown in Figure 1, only one band with expected size was detected in the Southern hybridization, which confirms that the *OsDof12 *gene is a single copy in rice genome.

### Characterization of rice *OsDof12 *and *OsDof12os *genes

Four full-length cDNAs (006-303-F03, 001-035-D05, J013026L11 and J013089G12) associated with *OsDof12 *were found by searching the database of full-length cDNA clones from japonica rice [28]. Of the four clones, 001-035-D05, J013026L11 and 006-303-F03 had sequences identical to each other and were transcripts of *OsDof12*, whereas J013089G12, termed as *OsDof12os *in this study, was transcribed from the opposite strand of *OsDof12*. To confirm the sequences of *OsDofl2 *and *OsDofl2os*, we performed RT-PCR experiments on RNA samples extracted from the whole rice plant to amplify full-length cDNAs of *OsDofl2 *and *OsDofl2os *with the primer pairs (Table 1). The sequencing results indicates that the *OsDofl2os *and *OsDofl2 *has no alternative splicing, and that the sequences of *OsDofl2os *and *OsDofl2 *we got are in good agreement with J013089G12 and 001-035-D05, respectively.

**Table 1:** List of primers

Name	Sequence
OsDof12-F	5'-CTTCCAAGAGGGATCTTGAC-3'
OsDof12-R	5'-CTTGCTCTCCCTATCTTTCTC-3'
OsDof12os-F	5'-CCACTGTGTTGAAGGTCCTG-3'
OsDof12os-R	5'-GCTAGAGATCAGAACATGTGG-3'
OsDof12-FL-F	5'-GTGCGAATGAAAAGATTTCAAG-3'
OsDof12-FL-R	5'-CATATTATAAGGGGAACTTGCTC-3'
OsDof12os-FL-F	5'-GAGGCGTTGTTCAAGTCAAG-3'
OsDof12os-FL-R	5'-CAGATTTAACCAACACAAATTGC-3'
Probe C-F	5'-ATCGGCCGCTTCCCATTTCC-3'
Probe C-R	5'-GCATCCTCTGCCATGATCTG-3'

**Figure 2:**
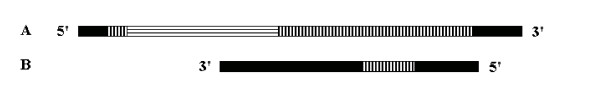
**(A) Organization of *OsDofl2 *transcripts**. The structures of *OsDofl2 *transcripts, deduced from our results, are displayed. **(B) Organization of *OsDofl2os *transcripts**. The putative structures of the *OsDofl2os *transcripts, deduced from our results, are displayed. Rectangles filled with horizontal lines indicate the intron. Rectangles filled with vertical lines represent the longest open reading frames. The black bars represent 5'-or 3'-untranslated regions.

To explicate the organization of sense and antisense *OsDof12 *transcripts, the structures of sense and antisense *OsDof12 *transcripts are shown in Figure 2. *OsDof12 *transcript contains two exons and one intron of 945 bp in size. The longest ORF encodes a predicted Dof protein of 440 amino acids with a Dof domain at its N-terminal regions. The *OsDofl2os *transcript is 1496 bp in size with no intron, and it encodes a protein of 104 amino acids. The OsDofl2os does not match any known proteins in the database. It is unclear whether it fulfils any role in the regulation of *OsDof12 *expression. The *OsDof12os *transcript not only shares a 1222 bp overlap with the *OsDof12 *transcript, but also 274 bp with the intron of *OsDof12*. According to the patterns of exon-intron structure, the *OsDof12 *and *OsDof12os *form a typical sense-antisense transcript pair.

### Expression patterns of the sense-antisense *OsDof12 *transcripts in different tissues and developmental stages

**Figure 3:**
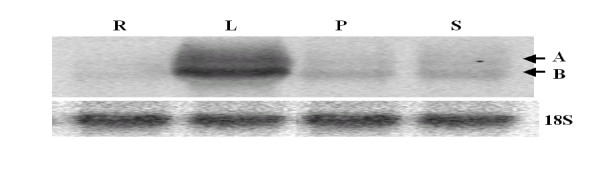
**Northern blotting analysis of rice *OsDof12 *and *OsDof12os *expression in different tissues**. Each lane was loaded with 10 μg total RNA isolated from leaves (L), roots (R), panicles (P) and stems (S) at panicle initiation stage. The blotting was hybridized with probe C, 18s rRNA (18S) was used as a loading control. A and B represent *OsDof12 *and *OsDof12os*, respectively.

Northern blotting with the probe C revealed two bands corresponding to the *OsDofl2 *and *OsDofl2os *transcripts, further analysis with the single-strand probes of *OsDof12 *and *OsDof12os *confirmed that the upper and lower bands were *OsDof12 *and *OsDof12os*, respectively. As shown in Figure 3, the basal expression of *OsDof12 *and *OsDof12os *transcripts was not tissue-specific, with the highest expression in young leaves, followed by stems, young panicles and roots. In addition, the *OsDof12 *and *OsDof12os *transcripts were co-expressed in the same tissues, with the expression of *OsDof12os *being higher than that of *OsDof12*.

**Figure 4:**
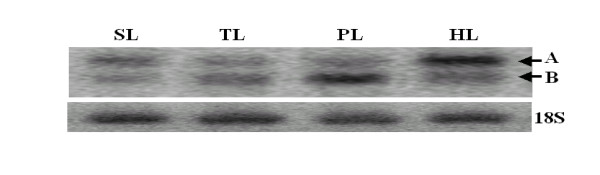
**Northern blotting analysis of *OsDof12 *and *OsDof12os *expression during different developmental stages**. Each lane was loaded with 10 μg total RNA isolated from rice leaves at seedling (SL), tillering (TL), panicle initiation (PL) and heading (HL) stages. All lanes were hybridized with probe C and then with a 18s rRNA (18S) as a loading control. A and B represent *OsDof12 *and *OsDof12os*, respectively.

To further analyze the expression patterns of the *OsDof12 *and *OsDof12os *transcripts, we also examined *OsDof12 *and *OsDof12os *transcripts in rice leaves at four developmental stages: seedling, tillering, panicle initiation and heading stages. Northern blotting analysis showed that the expression of *OsDof12 *and *OsDof12os *transcripts fluctuated through different developmental stages (Figure 4). As for *OsDof12 *transcript, the highest expression occurred at heading stage, and the lowest expression at tillering stage. Being different from *OsDof12*, The highest and lowest expression of *OsDof12os *was at panicle initiation and seedling stages, respectively. In addition, *OsDof12 *and *OsDof12os *exhibited inverse expression patterns at rice different developmental stages. For example, the expression of *OsDof12 *was higher than that of *OsDof12os *at seedling and heading stages, but lower than that of *OsDof12os *at tillering and panicle initiation stages. The inverse expression profiles of the *OsDof12 *and *OsDof12os *transcripts suggest that they are reciprocally regulated in rice development.

### The expression patterns of *OsDof12 *and *OsDof12os *under drought, ABA and dark treatments

**Figure 5:**
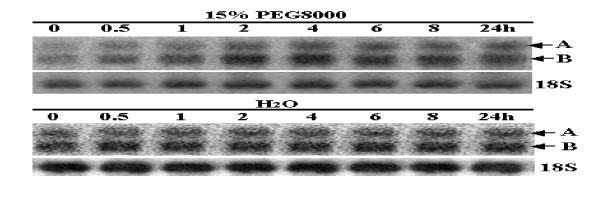
**Northern blotting analysis of *OsDof12 *and *OsDof12os *expression under drought treatment**. Each lane was loaded with 10 μg total RNA isolated from rice leaves that were exposed to water or 15% PEG8000 treatment for 0, 0.5, 1, 2, 4, 6, 8 and 24 h. All lanes were hybridized with probe C, 18s rRNA (18S) was used as a loading control. A and B represent *OsDof12 *and *OsDof12os*, respectively.

Dof proteins in plants have been reported to participate in the regulation of gene expression in diverse plant-specific biological processes [29]. Thus we detected the expression levels of the *OsDof12 *and *OsDof12os *transcripts in the rice plants treated with drought, ABA and dark. Both *OsDof12 *and *OsDof12os *transcripts in the leaves showed rapid and strong induction by drought stress (Figure 5). The induction was observed as early as 0.5 h after drought treatment, whereas, in the mock experiment, the *OsDof12 *and *OsDof12os *transcripts were not induced by water. The expression of the two transcripts reached the peak at 2 h, and then the expression levels remained constant until 24 h. Thus, during the drought treatment, the *OsDof12 *and *OsDof12os *transcripts were proportionally co-regulated and increased with time. Interestingly, the expression levels of the *OsDof12os *transcripts were always higher than those of *OsDof12*.

**Figure 6:**
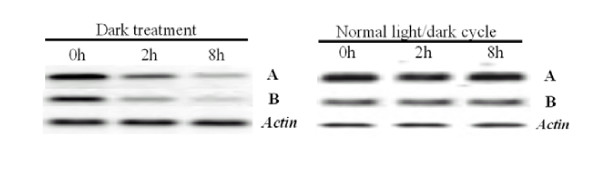
**RT-PCR analysis of *OsDof12 *and *OsDof12os *expression under dark treatment and normal light/dark cycle**. Plants were grown under normal light/dark cycle, and then switched to the dark. Leaves from dark treatment and normal light/dark cycle were harvested at 0, 2 and 8 h. A and B represent *OsDof12 *and *OsDof12os*, respectively. *Actin *was used as a control.

We also investigated the expression patterns of the *OsDof12 *transcript pair under ABA and dark treatments by RT-PCR analysis. Neither of the *OsDof12 *transcript pair was responsive to the ABA treatment (data not shown). In contract, the dark treatment had a great effect on the expression of the two transcripts. As shown in Figure 6, when the rice plants were moved from normal condition to complete darkness, the expression of *OsDof12 *and *OsDof12os *were inhibited by the dark treatment. Different from the drought treatment, the sense-antisense *OsDof12 *transcripts were decreased with time, and the expression levels of the *OsDof12 *transcript were higher than those of *OsDof12os*.

### Effect of the OsDof12 and OsDof12os's promoters on their expression

**Figure 7:**
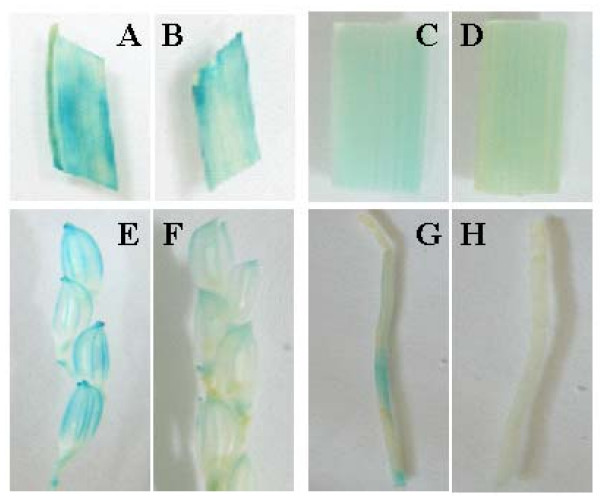
**Expression patterns of GUS in *Pro*_*OsDof*12_-*GUS *and *Pro*_*OsDof*12*os*_-*GUS *transgenic lines**. A, C, E, and G are respective expression patterns of GUS in leaves, stems, panicles and roots of *Pro*_*OsDof*12_*-GUS *transgenic lines at heading stage. B, D, F and H are respective expression patterns of GUS in leaves, stems, panicles and roots of *Pro*_*OsDof*12*os*_-*GUS *transgenic lines at heading stage.

As described above, the sense-antisense *OsDof12 *transcripts showed reciprocal or co-regulated expression patterns. We speculated that the expression patterns of two transcripts might be attributed to their promoters. To test the hypothesis, the vectors containing the GUS reporter gene under the respective control of the *OsDof12 *and *OsDof12os *promoters was used to transform the rice plants. At least 10 independent transgenic lines were analyzed at the heading stage. In the independent *Pro*_*OsDof*12_*-GUS *transgenic lines, the GUS staining was apparently observed in the leaves, stems, roots and panicles. Compared with the GUS staining in the *Pro*_*OsDof*12_*-GUS *transgenic lines, the GUS staining was weaker in leaves, stems and panicles in the independent *Pro*_*OsDof*12*os*_-*GUS *transgenic lines (Figure 7). Though GUS staining was not observable in roots in independent *Pro*_*OsDof*12*os*_-*GUS *transgenic lines, we did detect the *GUS *expression in roots by RT-PCR analysis (data not shown). As we expected, the GUS activities in *Pro*_*OsDof*12_*-GUS *and *Pro*_*OsDof*12*os*_-*GUS *transgenic plants were consistent with the RNA expression patterns of *OsDof12 *and *OsDof12os*, respectively.

## Discussion

The abundance, ubiquity, structural organization and other properties of overlapping transcripts suggest that they may play important roles in regulating gene expression, though the nature of the roles is unclear. In bacterial cells, some overlapping sense-antisense pairs display reciprocal expression patterns under a variety of conditions. In contrast to bacterial cells, increased expression of one member of a sense-antisense pair in eukaryotic cells is frequently not accompanied by a reciprocal decrease of the other, though the reciprocal relationship of the sense-antisense pair has been reported in certain cases [30,31]. Katayama et al. assessed possible regulatory interactions between sense-antisense transcript pairs by monitoring the correlation of expression with time. In that experiment, the sense- antisense pairs, whose expressions were substantially increased or decreased during the activation of bone marrow-derived macrophages by bacterial lipopolysaccharide (LPS), were selected for further analyses their expression by RT-PCR over 24 hours after activation of macrophages with LPS. Among the seven sense-antisense pairs showing various patterns of reciprocal regulation, three showed proportional co-regulation, where both members of the sense-antisense transcript pair decreased with time; Two pairs showed reciprocal regulation, where one transcript concentration was induced while the other declined in response to LPS. In other two sense-antisense pairs, the paired transcripts showed no obvious connection in their expressions [32]. In present study, the sense and antisense *OsDof12 *transcript pair were co-expressed in different tissues, and showed reciprocal regulation at different developmental stages. In addition, under the drought and dark treatments, the sense-antisense *OsDof12 *transcripts showed proportional co-regulation with time. Under the drought treatment, the expression of the transcript pair increased with time. In contrast, the expression of both transcripts decreased with time under the dark treatment. These results indicate that the expression of one member may have an effect on the other.

Although the abundant antisense transcripts have been identified, the roles of antisense transcripts are still elusive. However, pertinent proposal and data strongly imply that antisense transcripts can regulation of sense transcripts expression. Cis-antisense transcripts may regulate the expression of overlapping genes by competing for (or sharing) transcriptional factors/common transcriptional factor binding sites. For example, the result from DNase I footprinting suggested that imprinted Igf2R and Air promoters appear to share common cis-regulatory elements [33]. Sharing of common trans-acting factor may lead to co-expression of overlapping transcripts, whereas negative correlation is expected if distinct transcriptional factors compete for overlapping binding sites. This model is similar to that proposed for non-overlapping divergently transcribed gene pairs, which represent more than 10% of the genes in the human genome [34]. According to the model mentioned above and the GUS expression patterns in the Pro_*OsDof*12_*-GUS *and Pro_*OsDof*12*os*_-*GUS *transgenic rice, we speculated that the expression patterns of the sense-antisense *OsDof12 *transcripts might be relative to the common cis-regulatory elements shared by their promoters. We analyzed the cis-regulatory elements in the two predicted promoter regions of *OsDof12 *and *OsDof12os *by the database of PLACE [35], and the results showed that the promoter regions of the *OsDof12 *and *OsDof12os *each possessed 74 classes of cis-regulatory elements. Interestingly, among their respective 74 classes cis-regulatory elements, the predicted *OsDof12 *and *OsDof12os *promoters share 53 classes. Thus, the correlated expression patterns of *OsDof12 *and *OsDof12os *might be attributed to the basically common nature of the two promoter regions. To test this speculation, the accurate promoter regions of the sense and antisense *OsDof12 *should be identified, and the GUS activity of different deletions of the *OsDof12 *and *OsDof12os *promoters should also be analyzed.

Due to the sequence complementarity, a pair of co-expressed sense-antisense transcripts within the same cells would be possible to form double strand RNAs (dsRNAs), which in turn would lead to the generation of siRNAs [6,36]. This suggestion is supported by recent studies provided two examples of nat-siRNAs with an important role in regulating cis-NAT expression in response to abiotic and biotic stress in *Arabidopsis *[22-25]. Recently, Lu et al. found a unique class of nat-miRNAs, which was derived from natural cis-antisense transcript pairs, could induce mRNA cleavage in the middle of their complementary sites [37]. However, some researchers have argued against siRNA-mediated RNA cleavage as a model of antisense regulation [38-40]. Mohammad et al. showed that RNA interference is not involved in natural antisense mediated regulation of gene expression in mammals [38]. In Arabidopsis, Jen et al. proposed that the antisense transcript expression might mainly induce the alternative splicing or polyadenylation rather than induce the formation of dsRNA resulting in a predominant RNA degradation [39]. In addition, Henz et al. also suggested that there is a trend toward anti-correlated expression of cis-NAT pairs in *Arabidopsis*, but currently available data do not produce a strong signature of small RNA-mediated silencing for this process [40]. In the current case, although the *OsDof12 *and *OsDof12os *transcripts share complementary sequences and show reciprocally expression patterns, we cannot provide a clue to small RNA molecules related to the sense and antisense *OsDof12 *transcripts by the small RNA Northern analysis (data not shown). No small RNA was found relative to the sense and antisense *OsDof12 *transcripts by searching the rice small RNA database [41]. In addition, the decay of either *OsDof12 *or *OsDof12os *transcripts also was not detected by the 5'-RACE assay (data not shown). Considering that the co-expression of sense and antisense transcripts within the same cells is prerequisite to produce small RNA molecules, we have tried RNA FISH to see whether the *OsDof12 *and *OsDof12os *transcripts are co-expressed within the same rice cells, but we failed to do so. To our knowledge, it is still a difficult task to perform RNA FISH in plant cells. Therefore, whether the dsRNA- or siRNA-mediated RNA cleavage is involved in the *OsDof12 *and *OsDof12os *transcripts remains a question to be answered.

Although NATs exist widely in eukaryotes, most studies have only focused on the phenomena in general by globally analyzing the expression patterns of the sense and antisense transcript pairs in one organism with microarray. The exact mechanisms of antisense regulation in higher eukaryotes are largely unclear. It might be expected that there exists a balance between the expression of sense and antisense transcripts under normal conditions, which would be modulated by antisense regulation to adapt to different developmental and physiological stages or conditions. Disruption of the normal balance by mutating an associated sense or antisense gene or a regulatory element of either one may lead to disorders, as has been observed in *Neurospora crassa *[42]. Thus, the study of how natural antisense transcripts regulate gene expression is of great interest not only for gaining new insights into mechanisms of gene expression but also for better understanding the mechanisms in common and in difference between the plant and animal genomes.

## Conclusion

*OsDof12os *overlaps with both the exonic and intronic regions of *OsDof12*, so the *OsDof12 *and *OsDof12os *transcripts are a typical sense-antisense transcript pair. Being co-expressed, the sense-antisense *OsDof12 *transcripts showed no tissue-specific in general. During rice different developmental stages, the *OsDof12 *and *OsDof12os *transcripts exhibited reciprocal expression patterns. Interestingly, the expression of both genes was significantly induced under drought treatment and inhibited by dark treatment. The expression analyses of GUS in transgenic plants indicated that the promoters of *OsDof12 *and *OsDof12os *could explain their expression patterns. These results provide new insights into the organization of the rice *OsDof12 *locus and the expression patterns of the *OsDof12 *and *OsDof12os *transcripts.

## Materials and methods

### Plant materials and drought, ABA and dark treatments

Plants of the rice (*Oryza sativa*) variety, Nipponbare, were grown in a paddy field under natural conditions. Root, stem, young panicle, leaf tissues at panicle initiation stage and leaf tissues from four developmental stages (seedling, tillering, panicle initiation and heading stages) were harvested, frozen in liquid nitrogen, and stored at -80°C for further analysis.

Plants at the tilling stage were treated with drought (15% PEG8000). Leaf tissues from drought-treated and control plants were harvested after 0, 0.5, 1, 2, 4, 6, 8 and 24 h drought treatment, immediately frozen in liquid nitrogen, and stored at -80°C for RNA Gel-Blot Analysis.

Plants growing under normal light/dark cycle at seedling stage were moved to complete dark and treated with 100 μm ABA, respectively. The leaves from the ABA, dark-treated and control plants were harvested at 0, 2 and 8 h, immediately frozen in liquid nitrogen for RT-PCR analysis.

### Total RNA extraction and RT-PCR analysis

Total RNA was extracted from different tissues of *O. sativa *using RNeasy plant mini kits (Qiagen). RNA samples were treated with 20 units RQ1 DNase (RNase free) in the presence of 40 units of RNase inhibitor at 37° for 2 h. After extraction twice with phenol, the RNA was precipitated with ethanol and then dissolved in RNase-free double-distilled water.

All RT-PCR experiments described in this section were reproduced at least three times using independent cDNA preparations. Altogether, four pairs of PCR primers were designed to amplify the constitutive expression genes in the whole plant (Table 1). Using total RNA as template, reverse transcriptions of first strand cDNA were performed with SuperScript II reverse transcriptase (200 units; Invitrogen). PCR conditions were as the following: 5 min at 94°C, 25 or 30 cycles of 30 seconds at 94°C, 1 min at 56°C and 2 min at 72°C, and 10 min at 72°C. The RT-PCR products were analyzed by electrophoresis in 1.5% agarose gels. In addition, the RT-PCR products were further cloned into T-easy vector for sequencing analysis.

### DNA and RNA gel-blot analysis

Total genomic DNA was isolated from the leave tissues of *O. sativa*. Genomic DNA (10 ug) was digested with HindIII and EcoRI, respectively. The digested DNA was fractionated by electrophoresis in 0.8% agarose gel overnight. After depurinating in 0.25 N HCl, DNA fragments were transferred to Hybond-N1 membranes (Amersham).

Northern analyses were performed after removing DNA using RNase-free RQ1 DNase. Approximately 10 ug of total RNA per lane was electrophoresed on 1% agarose-formaldehyde gels and capillary blotted onto Hybond-N1 membranes (Amersham).

The probes used in Southern and Northern analyses were amplified with primer C (Table 1), then cloned into pGEM-T Easy vector and labeled with α-^32^P-dCTP using Random Primer DNA Labeling Kit (Takara). Two riboprobes were labeled by annealing the respective primers to total RNA and labeling with α-^32^P-CTP using the riboprobe in vitro transcription systems (Promega).

Southern or Northern hybridization conditions were the same. The membranes were incubated in standard prehybridization solution at 65°C for 6 h and then hybridized with α-^32^P-labeled probe at 65°C for 12 h. Following hybridization and sequential washing, the radioactive membranes were then exposed to x-ray film.

### Histochemical GUS analysis and promoter analysis

According to the information of the *OsDof12 *and *OsDof12os *full-length cDNA and rice genome sequence, the promoter regions of *OsDof12 *and *OsDof12os *were predicted by software. The *Pro*_*OsDof*12_*-GUS *and *Pro*_*OsDof*12*os*_-*GUS *was created by ligating about 1.5-Kb promoter sequence into pCAMBIA1301 without the 35S promoter, respectively. Nipponbare was used as the plant material to obtain the transgenic lines containing *Pro*_*OsDof*12_*-GUS *and *Pro*_*OsDof*12*os*_-*GUS *by an *Agrobacterium tumefaciens*-mediated transformation method.

Transgenic lines bearing the *Pro*_*OsDof*12_*-GUS *and *Pro*_*OsDof*12*os*_-*GUS *were incubated at 37°C overnight with GUS staining solution (100 mM sodium phosphate buffer, 1 mM 5-bromo-4-chloro-3-indolyl-b-D-glucuronic acid, pH 7.2, 10 mM EDTA and 0.1% Triton) to detect GUS activity. Following GUS staining, samples were washed several times to extract chlorophyll using a graded ethanol series.

The cis-regulatory elements in the promoter regions of sense and antisense *OsDof12 *were predicted by PLACE [35]. The classes of cis-regulatory elements were defined as following: If the promoter region contains several same cis-regulatory elements (for example, S000265) in positive/negative strands, we defined them as one class of cis-regulatory element.

### Splicing pattern and coding potential evaluation of full length cDNAs and annotated genes

Splicing patterns of transcripts encoded by full-length cDNAs were obtained by aligning the cDNA sequences to the *Oryza sativa *genome using the sim4 program [43]. To evaluate the coding potential of full-length cDNAs, their corresponding genomic sequences (determined by BLAST and sim4 result) were extracted and screened by GeneScan [44].

## Abbreviations

ds RNA: double-stranded RNA; siRNA: small interfering RNA; NATs: Nature Antisense Transcripts; PCR: Polymerase Chain Reaction; RT-PCR: Reverse transcription polymerase chain reaction; ABA: Abscisic acid; GUS: GlucuronidaseDof: DNA-binding with one finger; bp: base pair; cDNA: complementary DNA; FISH: Fluorescence in situ hybridization; KOME: Knowledge-based Oryza Molecular biological Encyclopedia; LPS: Lipopolysaccharide; PLACE: Plant cis-acting regulatory DNA elements; RACE: Rapid Amplification of cDNA Ends and ORF: Open Reading Frame.

## Authors' contributions

DL designed and conducted the experiments, data analysis, and participated in drafting and editing the manuscript. CY and XL generated the transgenic rice. GJ constructed the *Pro*_*OsDof*12_*-GUS *and *Pro*_*OsDof*12*os*_-*GUS *vectors. LZ designed and supervised the study and wrote the manuscript. All authors read and approved the final manuscript.
